# Reference intervals and sources of variation of pressure pain threshold for quantitative sensory testing in a Japanese population

**DOI:** 10.1038/s41598-023-40201-w

**Published:** 2023-08-10

**Authors:** Hidenori Suzuki, Shu Tahara, Mao Mitsuda, Masahiro Funaba, Kazuhiro Fujimoto, Hiroaki Ikeda, Hironori Izumi, Kiminori Yukata, Kazushige Seki, Kota Uranami, Kiyoshi Ichihara, Norihiro Nishida, Takashi Sakai

**Affiliations:** 1 Department of Orthopaedics Surgery, Yamaguchi University Graduate School of Medicine, 1-1-1 Minamikogushi, Ube City, Yamaguchi 755-8505 Japan; 2 Pain Management Research Institute, Yamaguchi University Hospital, Yamaguchi, Japan; 3 Department of Rehabilitation, Yamaguchi University Hospital, Yamaguchi, Japan; 4 Faculty of Health Sciences, Yamaguchi University Graduate School of Medicine, Yamaguchi, Japan

**Keywords:** Musculoskeletal system, Health care, Medical research

## Abstract

Quantitative sensory testing (QST) is useful when analysing musculoskeletal pain disorders. A handheld algometer is most commonly used for pressure pain threshold (PPT) tests. However, reference intervals for PPTs are not elucidated. We assessed reference intervals of PPTs for QST in 158 healthy adult Japanese with no history of musculoskeletal or neurological problems. A handheld algometer was used to record PPT at five different assessment sites on the body: lumbar paravertebral muscle, musculus gluteus maximus, quadriceps, tibialis anterior muscle, and anterior talofibular ligament. Multiple regression analysis was performed to explore sources of variation of PPT according to sex, age, body mass index, UCLA Activity Level Rating, and Tegner Activity Score. Reference intervals were determined parametrically by Gaussian transformation of PPT values using the two-parameter Box-Cox formula. Results of multiple regression analysis revealed that age was significantly associated with PPT of lumbar paravertebral muscle and musculus gluteus maximus. In females, body mass index showed significant positive correlation with PPT of anterior talofibular ligament, and UCLA Activity Level Rating also showed significant positive association with tibialis anterior muscle and anterior talofibular ligament. Site-specific reference intervals of PPTs for Japanese are of practical relevance in fields of pain research using a handheld algometer.

## Introduction

In recent years, several published articles have shown that quantitative sensory testing (QST) is useful in the analysis of musculoskeletal pain disorders^1–6^. Based on the evidence from these studies, it is assumed that QST might be a useful tool in the analysis of the pathogenesis, classification, differential diagnosis, and prognosis of musculoskeletal pain^1–6^.

**Figure 1:**
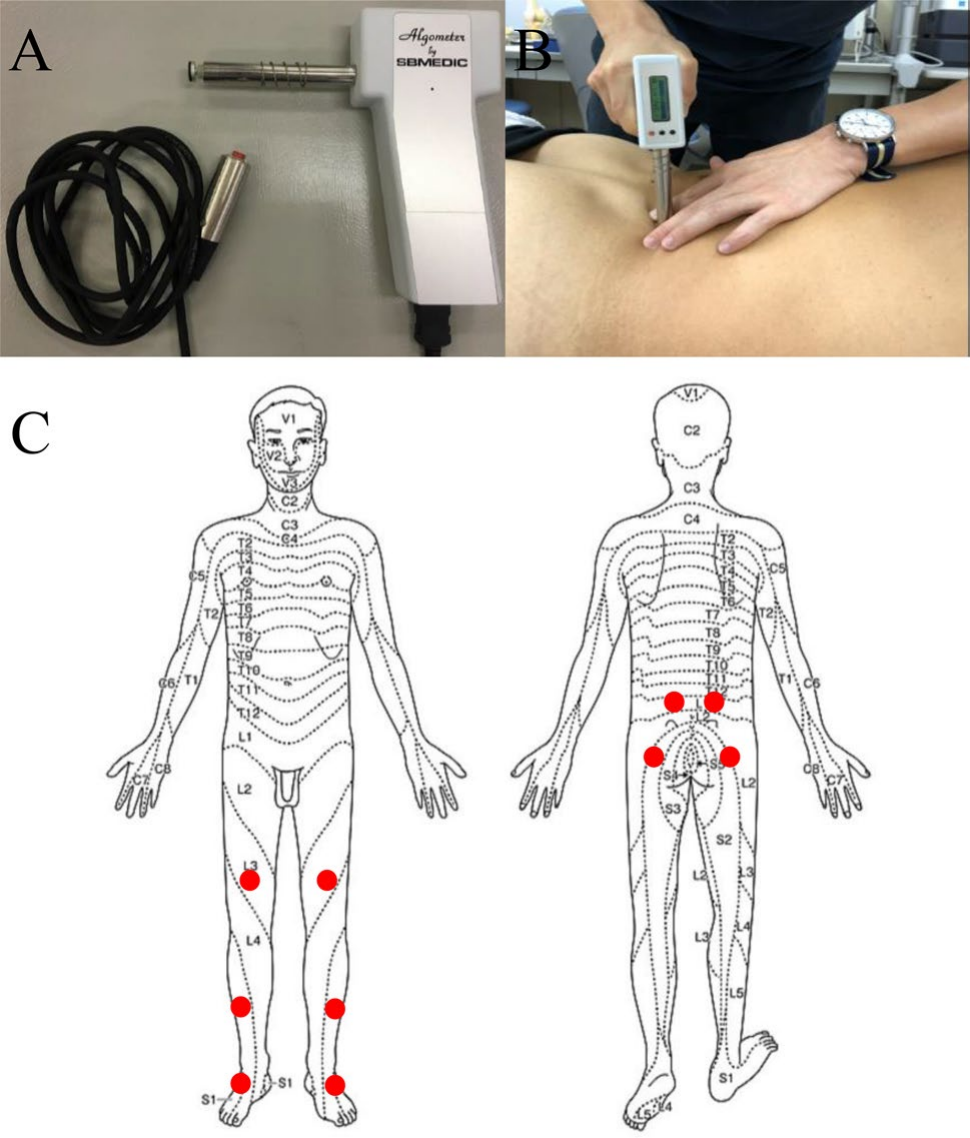
Handheld algometer and assessment of paravertebral muscles. (**A**) The handheld algometer used to assess pressure pain thresholds (PPTs). (**B**) Assessment of the paravertebral muscle. (**C**) Schematic of the body areas used for the analysis of PPTs. The assessment sites marked with red dots are as follows: (Top row) right and left lumbar paravertebral muscle at the level of iliac crest, 4 cm away from the spinal process; (Row 2) right and left musculus gluteus maximus, 5 cm below from iliac crest and 4 cm posterior to the posterior edge of the greater trochanter; (Row 3) right and left quadriceps, 10 cm proximal of the top of the patella; (Row 4) right and left tibialis anterior muscles, 5 cm distal and 3 cm lateral of the tibial tuberosity; (Bottom row) right and left of anterior talofibular ligament.

QST has become a common test in clinical neurophysiology units^1–3^. QST uses psychophysical tests defined as stimuli with predetermined physical properties based on specific measurement protocols for the analysis of somatosensory aberrations. QST measures responses to sensory stimuli and can be used to assess somatosensory system function, the measurement of altered peripheral and/or central pain sensitivity, and descending pain modulation^4,5,7^. Low back pain (LBP) is the most common musculoskeletal condition that evolves into chronic problems^8,9^. LBP patients with lumber disc herniation and/or lumbar spinal stenosis have also lower limbs pain because of the neurological symptoms^5^. L1 spinal nerve helps to move hip muscles. L2, L3 and L4 spinal nerves provide sensation to the front part of thigh and inner side of lower leg. L5 spinal nerve provides sensation to the outer side of your lower leg, the upper part of your foot and the space between your first and second toe. This nerve also controls foot and toe movements. Sciatic nerve from lumbar spine starts in rear pelvis and runs down the back of leg, ending in foot^5,8,9^. Therefore, we need to examine QST on the back, buttocks, femur, crus, and ankle in LBP patients (Fig. 1). However, the reference intervals of QST for LBP and lower limb radiculopathy are not available^5^.

A handheld algometer (Somedic, Hörby, Sweden) mounted with a 1-cm^2^ probe is the most common algometer used to evaluate PPT (Fig. 1)^3,5,10,11^. No statistically-based reference interval (RI) determined from well-defined healthy subjects is available for use in QST assessment in Japanese patients with musculoskeletal pain. For this reason, establishment of an appropriate RI has been in dire need for detecting any pathological changes in QST measurements^5^. For this reason, reference interval data are needed for QST protocols to assess musculoskeletal pain disorder.

Chronic LBP has a major impact on a patient’s quality of life^8,9^. In this study, we measured PPTs at points on the back, buttocks, femur, crus, and ankle in healthy Japanese to objectively assess pain intensity for future pain assessment in patients with LBP. The aim of this study was to investigate the reference interval of PPTs, which is defined as the central 95% value of PPTs measured at various assessment sites in healthy individuals. In addition, possible factors causing variation of PPT measurements were investigated, such as sex, body mass index (BMI), daily activity, and sports activity in healthy Japanese. We also discuss the characteristics and the cautions when using Japanese PPT data for widespread use of PPTs in LBP treatment in general clinics.

## Results

### Factors affecting variation of PPT measurements

Results of multiple regression analysis (MRA) are shown in Table 1 after setting a threshold of practical significance (effect size) as |r_p_| ≥ 0.3. Age showed a significant association with PPT-PVMin the males and with PPT-PVM and PPT-MGM in the females. In addition, BMI showed a significant correlation with PPT-TLin the females. ALR, which reflects daily activities, also showed significant association with PPT-TA and PPT-TL in the females^12^.

**Table 1:** Sources of variation of PPTs evaluated by multiple regression analysis.

	Exp para	n	R	Age	BMI	ALR	TAS
Males	PPT-PVM	85	0.521	**0.542**	0.055	0.235	0.086
	PPT-MGM	85	0.317	0.238	0.142	0.167	0.075
	PPT-QC	85	0.465	0.268	0.290	0.027	0.192
	PPT-TA	85	0.374	0.287	0.177	0.136	0.103
	PPT-TL	85	0.316	0.151	0.185	-0.073	0.214
Females	PPT-PVM	73	0.571	**0.598**	0.138	0.173	0.197
	PPT-MGM	73	0.402	**0.440**	0.114	0.020	0.275
	PPT-QC	73	0.396	0.254	0.299	0.185	0.007
	PPT-TA	73	0.393	0.220	0.207	**0.319**	0.039
	PPT-TL	73	0.454	0.017	**0.388**	**0.361**	-0.133

Significant values are in bold.

*PPT* pressure pain threshold, *Exp* para experimental parameter, *BMI* body massindex, *ALR* UCLA Activity Level Rating, *TAS* Tegner Activity Score, *PVM* lumbarparavertebral muscle, *MGM* musculus gluteus maximus, *QC* quadriceps, *TA* tibialisanterior muscle, *TL* anterior talofibular ligament.

**Figure 2:**
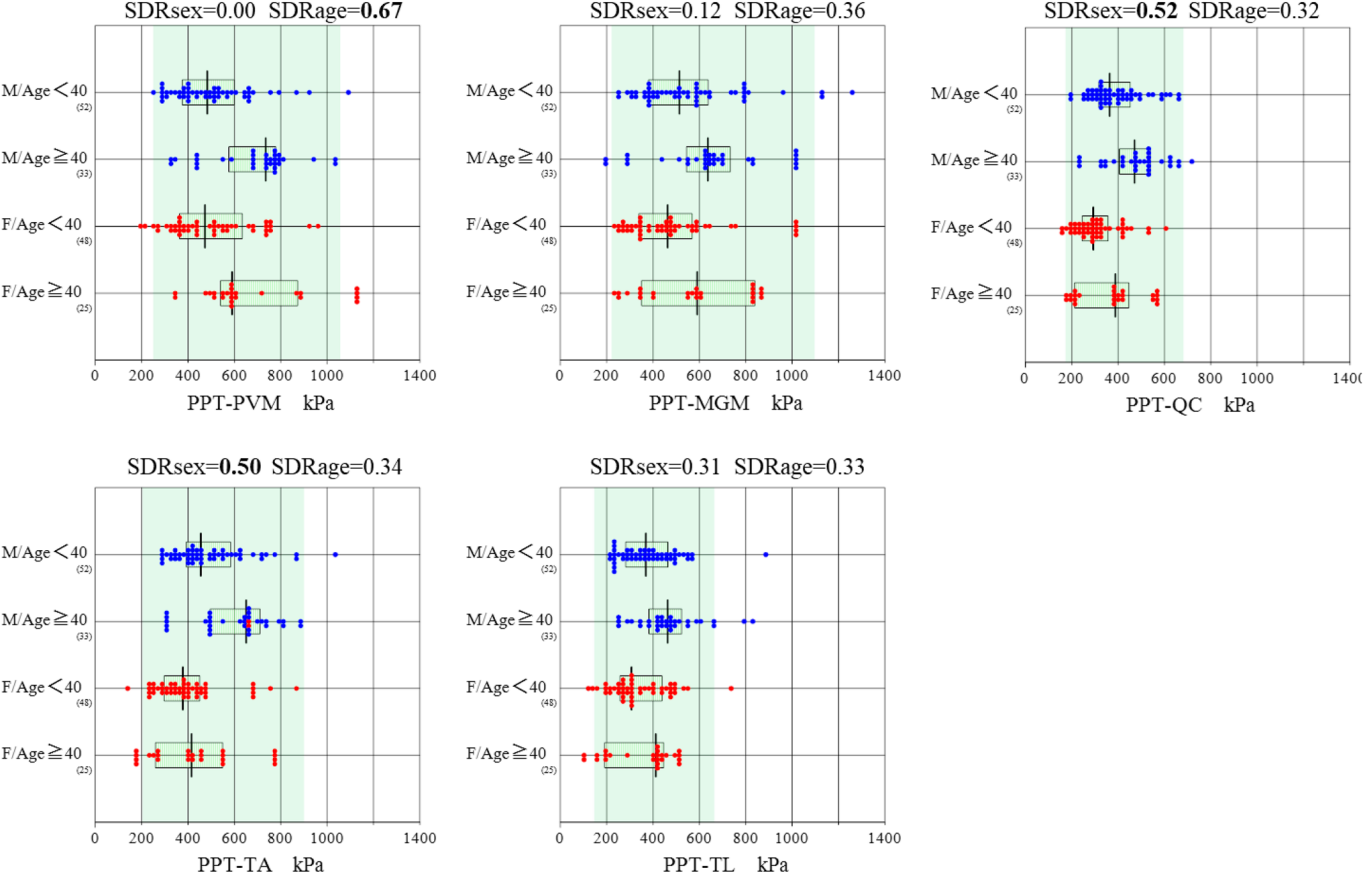
Sex and age-related changes in pressure pain thresholds (PPTs) at the five testing sites. Reference values (RVs) of PPTs recorded at the five sites (lumbar paravertebral muscle [PVM], musculus gluteus maximus [MGM], quadriceps [QC], tibialis anterior [TA], and talofibular ligament [TL]) were subgrouped by sex and age at 40 years. The box in the centre of each scattergram indicates the mid 50% range of RVs, and its central vertical bar represents the median. The data size of each subgroup is shown at the right bottom of the group labels. On the top of each graph, the magnitudes of the between-group differences by sex or age are shown as the SD ratio (SDR), SDRsex or SDRage, respectively. SDRs that exceeded the threshold of 0.4 are marked in bold. The background green shading indicates the reference interval determined from all RVs without partition by sex or age. The x-axis range was fixed at 0–1400 kPa for all graphs to show the test site dependency of the PPTs.

These findings are reflected in Fig. 2, which shows between-sex and between-age changes of PPTs at each assessment site. PPT-TL in the females positively correlated only with BMI (partial correlation coefficient: 0.388). PPT-TA and TL in the females were positively associated with ALR (0.319 and 0.361, respectively). There were no associations with PPTs andTAS, which reflected levels of sports activities^13,14^.

### Need for partitioning PPT reference values by sex and age

The above source-of-variation analysis by MRA revealed that there were significant sex and age differences in PPT depending on the assessment sites. To determine the need for partitioning reference values by sex and/or age, two-level ANOVA was performed. The magnitudes of between-sex and between-age variation were each calculated as a standard deviation ratio (SDR), SDRsex and SDRage, respectively^52^. By adopting 0.4 as a threshold for SDR, SDRsex^50^ was significant at the site of PPT-QC (0.520) and at that of PPT-TA (0.503), whereas SDRage was significant at the site of PPT-PVM (0.669) as shown in Table 2.

**Table 2:** Reference intervals of PPTs.

Item	Sex	Age	n	90% CI of LL		Reference interval			90% CI of LL		Variations of PPTs by sex and age	
				Low	High	LL	Me	UL	Low	High	SDRsex	SDRage
PPT-PVM	M/F	All	158	229	273	***250***	***539***	***1057***	990	1107	0.000	**0.669**
	M/F	< 40	100	216	264	**238**	**475**	**928**	845	1032		
	M/F	≥ 40	58	296	385	**324**	**665**	**1138**	1033	1245		
	M	All	85	235	302	268	548	1037	968	1118		
	F	All	73	201	281	240	526	1101	963	1218		
PPT-MGM	M/F	All	158	205	250	***222***	***519***	***1095***	1024	1185	0.119	0.357
	M/F	< 40	100	235	264	249	472	1090	945	1268		
	MF	≥ 40	58	115	250	155	614	1051	958	1134		
	M	All	85	189	265	225	564	1115	1000	1227		
	F	All	73	226	253	238	462	1087	966	1204		
PPT-QC	M/F	All	158	160	191	***174***	***366***	***682***	643	716	**0.520**	0.322
	M/F	< 40	100	162	199	179	337	632	557	686		
	M/F	≥ 40	58	37	200	83	441	681	634	726		
	M	All	85	193	236	**212**	**415**	**700**	663	744		
	F	All	73	154	178	**163**	**310**	**626**	564	676		
PPT-TA	M/F	All	158	166	214	***204***	***455***	***900***	849	943	**0.503**	0.336
	M/F	< 40	100	181	263	243	420	860	731	944		
	M/F	≥ 40	58	84	216	103	532	898	839	980		
	M	All	85	277	298	279	**514**	**932**	865	1000		
	F	All	73	141	202	169	**379**	**831**	719	907		
PPT-TL	M/F	All	158	126	169	***147***	***368***	***664***	627	740	0.313	0.328
	M/F	< 40	100	145	199	164	343	616	577	732		
	M/F	≥ 40	58	66	175	126	416	715	620	780		
	M	All	85	196	235	216	390	743	657	826		
	F	All	73	71	147	115	336	620	553	687		

*PPT* pressure pain threshold, *CI* confidence interval, *LL* lower level, *UL* upper level, *Me* medium, SDR standard deviation rate, *PVM* lumbar paravertebral muscle, *M/F* male and female, *M* male, *F* female, *MGM* musculus gluteus maximus, *QC* quadriceps, *TA* tibialis anterior muscle, *TL* anterior talofibular ligament.

*SDR threshold = 0.40, Unit; kPa.

### Derivation of assessment site-specific reference intervals for PPT

The reference interval of PPTs at each assessment site was calculated as the lower limit (LL: 2.5% point), median (Me: 50% point), and upper limit (UL: 97.5% point) as listed in Table 2. For partitioning of reference values by age, we arbitrarily set 40 years as a mid-age range for boundary. When SDRsex or SDRage exceeded the threshold of 0.40^49^, corresponding reference intervals (LL ~ Me ~ UL) were marked in bold. Otherwise, reference intervals without partitioning were shown in italics as the default. Notably, the range of reference intervals was generally low for PPT-QC, PPT-TA, and PPT-TL, implying that between-individual variations of PPTs are narrow. In contrast, the range was wider for PPT-PVM and PPT-MGM. These site-specific differences in the variability of PPT measurements are also apparent from Fig. 2.

## Discussion

QST is a formal variant of a time-honoured clinical examination technique in neurology. Currently, none of the neuropathic pain medications on the market have been developed based on prediction of treatment efficacy by QST. However, such an approach to the development of medicines has been encouraged by the European Medicines Agency^15,16^.

In the assessment of musculoskeletal conditions, clinicians often identify points of tenderness in superficial tissue^15,16^. The handheld algometer we used calculates PPT on superficial tissue that equate to one point of skin tenderness as found clinically when assessing a painful area^16–20^. It also includes measures of temporal summation by wind-up and documentation of dynamic mechanical allodynia^18–23^. When appropriate standards are applied, PPT can provide important and unique information about the functional status of the somatosensory system, which would complement already existing clinical methods^20–22^. Unfortunately, to our knowledge, reference intervals for the Japanese have never been reported using the handheld algometer for PPTs even though this measurement system is the most popular in the fields of pain research^3,5,18–22^. In addition, detection of PPT at the site of the most severe pain in patients with LBP was reported to be the most useful test in the assessment of hypersensitivity^24^.

Several studies of PPTs were reported previously in the study of LBP in western countries^1,5,15,17–22,24–40^. All of the studies used healthy control data to compare with the data of the patients with LBP^15,23–37^. PPT data using manual/electronic pressure algometry and cross-friction algometry in healthy control subjects from the lower back and QC or hip, gluteus maximus, or femur were reported. However, it was difficult to compare these studies’ results directly with our results because the algometry system itself was different from the instrument we used^5,25–27^.

In western countries, the data from healthy control subjects was reported at lumbar sites^5,28^. Lumbar PPT ranged from 299 to 628 kPa in the back area in these healthy control subjects^5^. However, the sample size was statistically too small to use the range as a reference interval for the PPT^5^. In the present study, we determined the reference intervals and sources of variation of the PPT for quantitative sensory testing by enrolling a larger number of healthy individuals, as shown in Table 2, which exceeded the minimum sample size of 120 recommended for determining reference intervals in the field of laboratory medicine^29^. The average values for all ages were as follows: PVM PPT-PVM, 539 kPa; PPT-MGM, 519 kPa; PPT-QC, 366 kPa; PPT-TA, 455 kPa; and PPT-TL, 368 kPa. We reviewed the previously published data in healthy control subjects measured by the instrument: The range of PPT at the lumbar area was 299–628 kPa, that at the gluteal areas was 535.9–863.97 kPa, and that at the lower leg was 321.8–771.5 kPa^5,15,23–38^. There were some differences between the articles depending on the volunteers’ backgrounds. However, these data may suggest differences in PPT in each race^37^: but more studies should be performed to confirm it.

The present study revealed the PPT at the PVM, MGM, QC, TA, and TL in healthy Japanese ranging in age from 19 to 59 years for the first time, to our knowledge (Table 2). It also revealed that the PPT at the PVM, MGM, QC, TA, and TL in healthy Japanese differed at each site, and these differences were influenced by sex, age, BMI, and ALR (Table 2). We show the characteristics of the PPT data for each site in Fig. 2. The variability of PPT-PVM and PPT-MGM was comparatively large, whereas that of PPT-QC, PPT-TA, and PPT-TL was small (Fig. 2, Table 2). These reference intervals should be used clinically with caution for patients with LBP.

Female participants showed higher pain thresholds for PPT-QC and PPT-TA compared to the male participants (Table 2). Females showed more tolerance than males for QST parameters, consistent with prior studies^1,39–42^. These sex differences in pain thresholds are unlikely to be due to peripheral factors such as innervation density and different central processing^1,41,42^.

In previous reports, obese individuals were more sensitive to pressure pain than individuals within a normal range of BMI^43^. Pain response varied according to subcutaneous body fat at different body sites^43^. However, PPT-TL in the females correlated only with BMI in the present study. Most of our participants were within the standard range of BMI, and therefore, we thought that our data showed a weak correlation between PPT and BMI only in the females.

PPT-TA and TL in the females were associated with ALR (correlation coefficients: 0.319 and 0.361, respectively). This result indicates that Japanese females with higher daily activity are more tolerant of lower leg pain. In addition, there was no association between PPT and the TAS, which reflects sports activities. We did not detect any correlation of PPT with sports and daily activity level in the healthy Japanese male volunteers. Previous articles revealed that exercise-induced hypoalgesia occurred following exercise. In addition, endocannabinoid levels were found to be elevated following exercise^44–46^. Our results showed a correlation between PPT and the questionnaire on activities only in the females of our study. We could not directly compare people with low activity to those with moderate or high activity because the participants basically all participated in moderate to slightly high activity levels of sports and daily activities, and no people with low activity were included.

The limitation of this study was relatively small sample size of healthy individuals recruited: n = 158. It was regarded as acceptable for determining the RIs without partitioning by sex and age. However, despite statistically significant between-sex and between-age differences in PPT, we could not reliably determine the RIs specific for each subgroup. It is certainly necessary to expand the scale of the study for better clinical usage of the RIs. The other limitation was that inter-rater reliability was not examined in this study. Therefore, we could only show the reference intervals for PPT in this study.

In summary, the present study determined the reference intervals of PPTs for the first time in healthy Japanese. In addition, we revealed that the PPTs at the PVM, MGM, QC, TA, and TL in healthy Japanese differed at each site and that these differences were influenced by sex, age, BMI, and ALR. Therefore, the above key points need to be taken into consideration when PPTs are measured in the assessment of patients with LBP. We hope that these data may become the reference intervals for the assessment of Japanese patients with LBP.

## Conclusions

In this study, we determined test site-specific reference intervals for pressure pain thresholds in quantitative sensory testing in a healthy Japanese population. In addition, we showed that sex- and age-related differences in pressure pain thresholds also depend on the site of assessment. Consequently, in the clinical assessment of patients with low back pain, it is necessary to take into considerations that reference intervals of pressure pain thresholds differ according to the site of assessment and that the levels of pressure pain thresholds are influenced by sex, age, body mass index, and UCLA activity level rating. These findings may be of practical relevance in the fields of pain research using pressure pain thresholds measured by the handheld algometer in Japan.

## Methods

### Subjects

Participants in this study included 158 healthy Japanese subjects (73 females, 85 males; age, 35.2 ± 12.7 years [mean ± SD]; BMI, 22.3 ± 3.03 kg/m^2^) with no history of musculoskeletal or neurological problems. Participants were medical staff, medical doctors, rehabilitation staff, medical students and their families in our institution. None of the participants had (1) ongoing pain problems, (2) circulatory disorders, (3) a history of metabolic disease or neuropathy, (4) current use of prescription medications, including analgesics, tranquilizers, antidepressants, or other centrally acting agents, (5) diagnosed mental health disorders, (6) current pregnancy, (7) liver or kidney disease, and (8) disorders involving the neuroendocrine system. The subjects were given a detailed written and verbal explanation of the procedures for measuring PPT, and all signed an informed consent form. This study was conducted in accordance with the Declaration of Helsinki and was approved by the Institutional Review Boards of Yamaguchi University (H2020-169) in May 2020.

### Experimental protocol

As the QST, the PPT was recorded 5 times at each of five pressure points (see below), and the average of the middle 3 scores, excluding the top and bottom score, was used for the data of pressure thresholds measured at each point (Fig. 1). A handheld algometer (Somedic, Hörby) mounted with a 1-cm^2^ probe (covered by a disposable latex sheath) was used to record the PPTs at 10 different locations on the body (Fig. 1). The investigator placed the handheld algometer on a site to be inspected and pressed against the tester in a vertical direction (Fig. 1). The investigator instructed the subjects to push the button by themselves when they felt slight pain. An interval of at least 20 s was kept between each assessment of a PPT. The PPT was defined for the subjects as “the time point at which the pressure sensation changed into pain.” Pressure was increased gradually at a rate of 30 kPa/s until the pain threshold was reached and the subject pressed a button^3,10,11,47^.

The assessment sites were as follows: (1) right and left lumbar paravertebral muscle (PVM) at the level of iliac crest, 4 cm away from the lateral side of spinal process; (2) right and left musculus gluteus maximus (MGM), 5 cm below from iliac crest and 4 cm posterior to the posterior edge of the greater trochanter; (3) right and left quadriceps (QC), 10 cm proximal of the top of the patella; (4) right and left tibialis anterior (TA), 5 cm distal and 3 cm lateral of the tibial tuberosity; and (5) right and left anterior talofibular ligament (TL). (1) and (2) assessments were in a prone position. (3), (4) and (5) assessments were in the supine position.

### Questionnaires

We evaluated daily activity with the UCLA ALR^12^, which is a single-item 10-level scale, ranging from level 10, representing a highly physically active person, to level 1, a person who is dependent on others and unable to leave home.

We evaluated sports activity with the TAS, which was developed by Tegner and Lysholm in 1985^13^. An activity level of 10–6 corresponds to participation in competitive and/or recreational sports, 5–1 corresponds to participation in recreational sports and heavy/moderate/light labour, and 0 is recorded for a person on sick leave or with a disability pension^13,14^.

### Statistical analyses

Summary values for numerical parameters are presented as the mean and standard deviation (SD). To explore sources of variation of PPTs, MRA was performed, separately for each sex. The PPT at each location was set as an objective variable and age, BMI, ALR, and TAS as the explanatory variables. The contribution of each variable to prediction of the level of PPT was expressed by the standardized partial regression coefficient (r_p_), which corresponds to a partial correlation coefficient and takes a value between − 1.0 and 1.0. The practical significance was set to |r_p_| ≥ 0.30 guided by the medium effect size of Cohen for correlation coefficients (0.3)^48^. To evaluate the need for partitioning PPT values by sex and/or age, two-level nested ANOVA was performed, in which age was partitioned at 40 years. Variations of PPT by sex and age were computed as SDs, SDsex and SDage, respectively. The SDR was computed by dividing each by between-individual (residual) SD (SDind) as SDRsex = SDsex/SDind and SDRage = SDage/SDind, respectively. The threshold for SDR was set to 0.4^47^. Reference intervals were determined parametrically after Gaussian transformation of the PPT by use of the two-parameter Box-Cox formula^49,50^:

$$X=\frac{{\left(x-\alpha \right)}^{\lambda }-1}{\lambda },$$

where *x* and *X* denote a PPT value before and after transformation, and the parameters λ and α represent power and shift (or a start), respectively, of data distribution. Details of the computation are as described elsewhere^51,52^. All data were analysed using StatFlex Ver. 7 for Windows (Artec, Osaka, Japan; https://www.statflex.net/)^51,52^.

## Data Availability

The authors confirm that the data supporting the findings of this study are available within the article.

## Abbreviations

QST: Quantitative sensory testing
PPT: Pressure pain threshold
PVM: Pressure pain threshold
MGM: Musculus gluteus maximus
QC: Quadriceps
TA: Tibialis anterior muscle
TL: Anterior talofibular ligament
MRA: Multiple regression analysis
BMI: Body mass index
ALR: UCLA activity level rating
TAS: Tegner Activity Score
LBP: Low back pain
PPT-PVM: Pressure pain threshold at the area of lumbar paravertebral muscle
MGM: Pressure pain threshold at the area of musculus gluteus maximus
PPT-TL: Pressure pain threshold at the area of anterior talofibular ligament
PPT-QC: Pressure pain threshold at the area of quadriceps
PPT-TA: Pressure pain threshold at the area of quadriceps
PPT-TL: Pressure pain threshold at the area of anterior talofibular ligament
